# Development and validation of a LC-MS/MS method for the quantification of the E-type prostanoid receptor 4 antagonist HTL0039732 in human plasma, for a Phase I/IIa clinical trial

**DOI:** 10.1080/17576180.2026.2712785

**Published:** 2026-08-06

**Authors:** Dan Astley, Svatopluk Svetlik, Shelby Barnett, Gareth J. Veal

**Affiliations:** a Newcastle University Centre for Cancer, Newcastle University, Newcastle-Upon-Tyne, UK; b Cancer Research UK Centre for Drug Development, London, UK

**Keywords:** HTL0039732, EP4, prostaglandin, LC-MS/MS, cancer, pharmacokinetics, validation

## Abstract

**Background:**

HTL0039732 (HTL) is a novel E-type prostanoid receptor 4 (EP4) antagonist for the treatment of advanced solid tumors. An analytical method was developed to facilitate the analysis of patient samples as part of an ongoing Phase I/IIa clinical trial.

**Methods and results:**

A LC-MS/MS method for quantifying HTL in human plasma was developed and validated per regulatory guidelines. The assay was selective, with a 2 ng/mL detection limit observed. It demonstrated good precision (CV ≤11.9%) and accuracy (86–101%), with linearity from 10 to 2000 ng/mL. Recovery was consistent, and no significant matrix, anticoagulant, or carryover effects were observed.

**Conclusions:**

A sensitive and selective method for measuring HTL in human plasma was successfully developed and applied to clinical samples, generating first‑in‑human pharmacokinetic data for HTL. The assay is now being utilized in an early‑phase clinical trial setting.

**Clinical trial registration:**

https://clinicaltrials.gov/study/NCT05944237.

## PLAIN LANGUAGE SUMMARY

We created a method to measure levels of a new cancer drug called HTL in plasma (a component of blood). We did this because the drug was being tested in humans for the first time. We needed to measure how levels of the drug changed over time in each patient receiving the drug. We conducted various experiments to check how our method performed. This was to ensure that our method was reliable, could measure HTL consistently and accurately, and that the drug was stable under storage conditions. We found that our method met these criteria. It was then used to measure HTL levels in samples from cancer patients in the clinic.

## 1. Introduction

Evasion of the immune system is one of the main hallmarks of cancer that allows malignant cells to thrive. These cells develop the ability to shield themselves from the host’s immune system by hijacking anti-inflammatory pathways that suppress the immune response in the tumor microenvironment [1]. Targeting components of immunosuppressive signaling pathways therefore represents an avenue for improving treatment.

A method of inducing immunosuppression that cancer cells are able to utilize involves secretion of programmed death-ligand 1 (PD-L1). This ligand binds to programmed death protein 1 (PD-1), a receptor expressed on the surface of T-cells. Activation of PD-1 by PD-L1 causes several inhibitory effects to T-cells, including decreased activation, proliferation and cytokine secretion, and an increase to the rate of apoptosis [2]. These effects make PD-L1 an attractive therapeutic target. Atezolizumab is a monoclonal antibody that binds to PD-L1 with inhibitory effects, preventing it from activating the PD-1 receptor, thus protecting T-cells and restoring their anti-cancer activity [3].

Another pathway that cancer cells are able to use to their advantage is the prostaglandin (PG) E2/E-type prostanoid receptor 4 (EP4) pathway. PGE2-mediated activation of the EP4 receptor induces immunosuppression within the tumor microenvironment by downregulating natural killer cell activation and dendritic cell maturation [4]. Additional suppression of T-cells is achieved by upregulation of myeloid-derived suppressor cells [5]. The synthesis of PGE2 is closely regulated by Cyclooxygenase-2 (COX-2), with overexpression of COX-2 and PGE2 being linked with increased EP4 signaling and downstream immunosuppressive effects [6].

The novel drug HTL0039732 (HTL) has been developed as a potent, specific antagonist of the EP4 receptor to be used in the treatment of advanced solid tumors. This drug is currently being investigated as a monotherapy and in combination with atezolizumab in eligible patients participating in a Phase I/IIa clinical trial, in collaboration with Cancer Research UK (CRUK).

The ongoing clinical study includes dose escalation (Phase I) and dose expansion (Phase IIa) components. Phase I was designed to assess the safety, tolerability, pharmacokinetics (PK), and preliminary efficacy of HTL, as a monotherapy (part A) and in combination with atezolizumab (part B), and to inform the recommended Phase II dose (RP2D) [7]. The dose‑expansion phase of the study has commenced, where patients will receive HTL in combination with atezolizumab or other approved immune checkpoint inhibitors. Further PK data will be generated in Phase IIa, and the activity of this novel compound will be explored in more focussed grouping by cancer type.

Incubation of HTL with human hepatocytes and toxicokinetic studies identified one major metabolite, an acyl glucuronide of HTL (M4). Due to the known safety risks associated with acyl glucuronides [8], and to determine whether this metabolite necessitates further characterization, its quantitative analysis was also necessary in the clinical trial.

The development of a quantitative assay to simultaneously determine plasma concentrations of HTL and M4 was required to facilitate the generation of PK study data. Liquid chromatography with tandem mass spectrometry (LC-MS/MS) was utilized for this purpose as an ideal technique for performing robust, selective, and sensitive quantification of small molecules. Here, we describe the development and validation of this method according to the appropriate international guidelines [9,10] and subsequent application to the analysis of samples from clinical trial participants.

## 2. Materials and methods

### 2.1. Standards and chemicals

**Figure 1.**
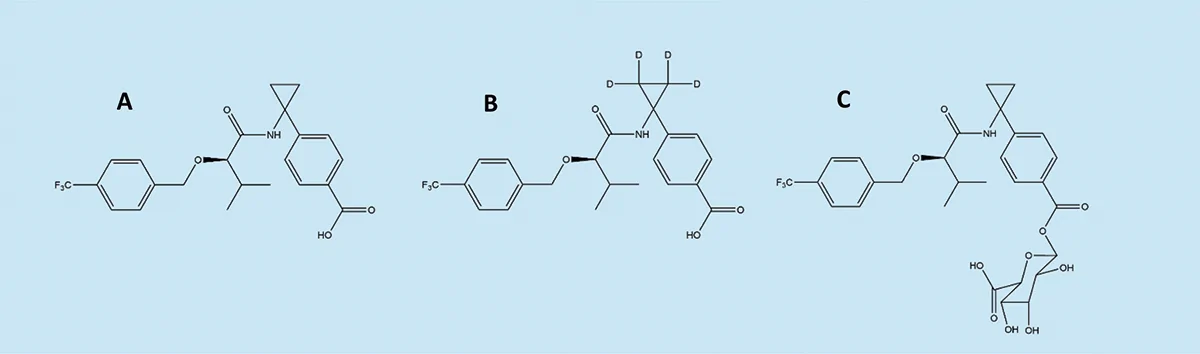
Chemical structures of (A) HTL (molecular weight 435.44 g/mol), (B) ^2^H_4_-HTL (molecular weight 439.47 g/mol), and (C) HTL-M4 (molecular weight 611.57 g/mol).

Analytical standards HTL0039732 (HTL), HTL0065658 (M4), and deuterium-labeled HTL (^2^H_4_-HTL) (Figure 1) were provided by Symeres (Nijmegen, Netherlands). Dimethyl sulfoxide (DMSO) was purchased from Honeywell Research Chemicals (Hanover, Germany). HPLC-grade acetonitrile and formic acid (FA) were purchased from Thermo Fisher Scientific (Leicester, UK). Whole blood and human plasma with sodium citrate were provided by the Blood Transfusion Center (Newcastle upon Tyne, UK).

### 2.2. Preparation of stocks, standards, and QCs

Stock solutions were prepared in DMSO by dissolving HTL, M4, and ^2^H_4_-HTL to 10, 5, and 1 mg/mL, respectively. Stocks for calibration standards and quality controls (QCs) were prepared independently to one another. The internal standard (IS) stock, ^2^H_4_-HTL, was serially diluted in acetonitrile to 10 µg/mL, then 0.1 µg/mL, which was used as the protein precipitation solvent during sample processing.

Calibration standards were prepared at seven concentration levels by spiking control plasma with HTL and M4. Spiking solutions were first prepared by serially diluting the DMSO stocks with acetonitrile. The spiking solutions contained both HTL and M4 at concentrations 50-fold higher than the plasma calibration points, allowing a spiking ratio of 50:1 (plasma:acetonitrile). The standard curve concentrations in plasma were 10, 20, 100, 400, 1200, 1800, and 2000 ng/mL for HTL and 5, 10, 50, 200, 600, 900, and 1000 ng/mL for M4.

Using the QC stocks, low, medium, and high QCs were prepared by spiking control plasma with HTL to 30, 800, and 1600 ng/mL and M4 to 15, 400, and 800 ng/mL within the same QC samples. Additional QCs were prepared at the lower limit of quantification (LLOQ) and the upper limit of quantification (ULOQ).

### 2.3. Sample processing

In a 0.5 mL polypropylene centrifuge tube, 50 µL of calibration standard, QC, blank, or patient plasma was combined with 250 µL of cooled acetonitrile containing ^2^H_4_-HTL at 0.1 µg/mL (or neat acetonitrile for plasma double blanks). Samples were immediately vortex mixed for 10 s, then centrifuged at 21,000 g for 5 min at 4°C. The resulting supernatant was transferred to a polypropylene insert within a HPLC vial prior to analysis. Both HTL and M4 were quantified using ^2^H_4_-HTL as an internal standard.

### 2.4. Liquid chromatography conditions

Chromatographic equipment included a Prominence 20 series HPLC system consisting of a SIL-20AC CR autosampler, two LC-20AD XR pumps, a CBM-20A communications module, and a CTO-20AC column oven (Shimadzu, Milton Keynes, UK). The column was a ZORBAX Eclipse Plus C18 Rapid Resolution HD 3.0 × 50 mm, 1.8 µm column coupled with a ZORBAX Eclipse Plus C18 Rapid Resolution HD UHPLC guard 3.0 × 5 mm, 1.8 µm (Agilent, Cheadle, UK), incubated to 30°C. Mobile phase (MP) A was 0.2% FA in water (v/v), and MP B was 0.2% FA in acetonitrile (v/v). The flow rate was 0.5 mL/min, and a step gradient was applied. Step 1: 30–95% B (0–2 min); Step 2: 95% B (2–3 min); Step 3: 95–30% B (3–3.1 min); Step 4: 30% B (3.1–4.5 min). The autosampler was cooled to 4°C, the injection volume was 10 µL, and the total run time was 4.5 min.

### 2.5. Mass spectrometer conditions

**Figure 2.**
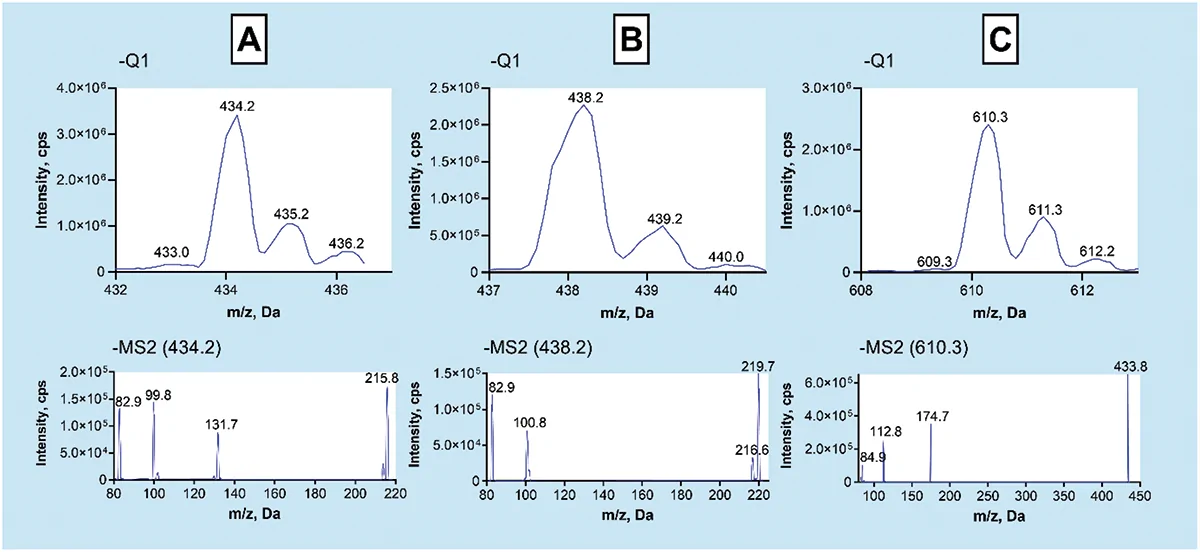
Full scan and tandem mass spectra showing precursor and product ion profiles of (A) HTL, (B) ^2^H_4_-HTL, and (C) HTL-M4 following direct infusion of mobile phase solutions containing each compound at 0.5 µg/mL into the TurboSpray ion source, operated in negative ion mode, at 7 µL/min.

Abbreviations: Cps: counts per second; Da: Daltons; MS2: negative mode tandem mass spectra; m/z: mass-to-charge ratio; Q1: negative‑mode full‑scan mass spectra.

An API 4000 triple quadrupole mass spectrometer (SCIEX, CA, USA) equipped with a Turbo Ion Spray probe was operated in negative electrospray mode. The settings were optimized by directly infusing individual solutions of HTL, M4, and ^2^H_4_-HTL, each prepared to 0.5 µg/mL in 50:50 mobile phase A: mobile phase B, at a flow rate of 7 µL/min. Automated compound optimization was used to determine the final multiple reaction monitoring (MRM) settings. A transition of 434.1 to 99.8 was used to quantify HTL; 610.3 to 433.8 was used to quantify M4; and 438.1 to 219.7 was used for ^2^H_4_-HTL (Figure 2). Source and gas settings were optimized manually to obtain maximum HTL signal. Ion Spray voltage was −4500 V, and temperature (TEM) was 500°C. Nitrogen was used for collision gas (CAD) at 7 psi and curtain gas (CUR) at 20 psi. Zero air was used for the nebulizing gas (GS1) and auxiliary gas (GS2), both of which were set to 50 psi.

### 2.6. Method validation

Method validation was performed in accordance with current United States and European guidelines on bioanalytical method validation [9,10]. The validation assessed limit of detection (LOD), limit of quantification (LOQ), selectivity, matrix effect, recovery, carryover, effect of anticoagulants, linearity and range, intra- and inter-day precision, dilution integrity, and stability. Acceptable accuracy was within 85–115% of the nominal concentration (or 80–120% for the lowest concentration). Acceptable precision was a coefficient of variation (CV) value of ≤15% (or ≤20% for the lowest concentration).

#### 2.6.1. Limits of detection and quantification

Standards containing HTL and M4 were prepared in plasma from 1 to 10 ng/mL then extracted with neat acetonitrile prior to LC-MS/MS analysis. Noise was first calculated as the highest observable point on the baseline in counts per second (cps). The signal-to-noise (S/N) ratio between the peak height of the standards and the baseline noise was calculated for each concentration. The LOD was determined as the concentration that gave a S/N ratio of 3:1. The LOQ was the concentration that gave a S/N ratio of 10:1. The LLOQ was further assessed by performing analysis of an extracted LLOQ sample (*n* = 8) together with a standard curve.

#### 2.6.2. Selectivity

The ability of the method to selectively detect HTL, M4, and ^2^H_4_-HTL was assessed by extracting blank plasma from six individual sources in duplicate with acetonitrile, then analyzing together with a standard curve. The responses (peak area, cps) for each analyte and ^2^H_4_-HTL were calculated at the expected retention times in all six batches of blank plasma. The mean response in each batch of plasma was acceptable if ≤20% of the mean response in the LLOQ for HTL and M4 and ≤5% for ^2^H_4_-HTL. Experiments to investigate the interference of potentially co-administered drugs were not carried out as part of the assay method validation.

#### 2.6.3. Matrix effect

Low, medium, and high QCs were each prepared in six different sources of plasma. Separate QCs at equivalent concentrations were prepared in 50:50 mobile phase A:mobile phase B. Matrix QCs (*n* = 1 per plasma source per concentration) and mobile phase QCs (*n* = 6 per concentration) were extracted with IS then analyzed. The matrix factor (MF) for each analyte and IS was calculated as the peak area in the plasma QC divided by the peak area in the solvent QC. IS-normalized MF was then calculated by dividing the analyte MF by IS MF.

#### 2.6.4. Recovery

Low, medium, and high QCs were each prepared in six different sources of plasma. The same plasma was also extracted with blank acetonitrile; then, the resulting supernatant was spiked with HTL, M4, and ^2^H_4_-HTL to concentrations equivalent to the extracted low, medium, and high QCs prepared in plasma. Recovery was calculated as the mean peak area in the matrix QCs as a percentage of the mean peak area in the supernatant QCs.

#### 2.6.5. Linearity and range

The standard curve range and linearity were assessed by extracting and analyzing a full set of calibration standards across five independent analytical runs. Linear regressions were performed using weightings of 1/x, 1/x^2^, 1/y, and 1/y^2^ to determine the highest mean coefficient of determination (r^2^) value across the five analyses.

#### 2.6.6. Intra- and inter-day precision

Control plasma from one individual donor was used to prepare LLOQ, low, medium, high, and ULOQ QCs. The QCs were extracted (*n* = 8 per concentration) and analyzed with a standard curve to assess intra-day precision. The inter-day precision was assessed by extracting and analyzing LLOQ, low, medium, and high QCs (*n* = 5 per concentration), with a standard curve, on five separate analysis days.

#### 2.6.7. Anticoagulant effect

Control blood was collected from one individual donor into Vacutainers (Becton Dickinson, Plymouth, UK) containing either Na-citrate, Li-heparin, or K_2_-EDTA. Plasma was generating by centrifuging whole blood at 872 g for 15 min at 4°C. Low, medium, and high QCs were prepared in plasma containing each anticoagulant. The QCs were extracted and analyzed (*n* = 3) together with a standard curve.

#### 2.6.8. Dilution integrity

A dilution QC (DQC) was prepared by spiking plasma to 20,000 ng/mL for HTL and 10,000 ng/mL for M4. The DQC was diluted with control plasma across four dilution factors (10-fold, 50-fold, 100-fold and 500-fold), then extracted and analyzed together with a standard curve.

#### 2.6.9. Stability

The stability of HTL and M4 in plasma was assessed at room temperature (RT) for 7 h and at −20°C for 12 months. The stability of the extracted samples under autosampler conditions (4 °C) was assessed for 24 h. Low and high QCs were prepared and immediately analyzed (*n* = 3), alongside a standard curve, to generate time zero (T0) data. The QCs were stored under the assessment conditions, then analyzed alongside freshly prepared standard curves. The percentage difference between the stability test and the T0 test was calculated.

#### 2.6.10. Carryover of the analyte and IS

Carryover was assessed by injecting a blank plasma sample immediately after the ULOQ QC (*n* = 2), alongside a standard curve. The mean response in the blank plasma was calculated as a percentage of the mean response in the lowest calibration standard. Acceptable carryover was ≤20% for HTL and M4 and ≤5% for ^2^H_4_-HTL.

### 2.7. Patient sample analysis

Blood samples from patients participating in the ongoing HTL Phase I/II clinical trial (ClinicalTrials.gov: NCT05944237) were collected in 3.5 mL citrate vacutainers pre-dose and 1, 2, 4, 6, 10, 24, 48, and 72 h following oral administration of doses between 80 mg and 640 mg of HTL. Trial participants were enrolled at five clinical sites: The Christie Hospital, Addenbrooke’s Hospital, Velindre Cancer Center, Guy’s Hopsital, and Clatterbridge Cancer Center. Plasma was generated from the blood samples by centrifuging for 10 min at 1,000 g and 4°C; the plasma was then transported on dry ice to Newcastle for analysis. Samples were stored at −20°C until they were processed and analyzed using the validated LC-MS/MS method. Non-compartmental analysis of the resulting data was performed to determine pharmacokinetic parameters, including C_max_, T_max_, half-life (T_1/2_), clearance (Cl/F), apparent volume of distribution (Vd/F), and area under the plasma concentration–time curve (AUC), using Phoenix WinNonlin, version 8.4 (Certara, MO, USA).

## 3. Results

### 3.1. LC-MS/MS

Operating in negative ionization mode, the deprotonated ions [M-1]^−^ gave the highest signal intensity for HTL, M4, and ^2^H_4_-HTL (Figure 2). The product ion of 99.8 was selected as a high-intensity and reproducible quantifier for HTL and demonstrated the highest signal-to-noise ratio. Source and gas settings were optimized to give maximum HTL signal. Optimized MRM scan settings are shown in Supplementary Figure S1.

Efficient chromatography was achieved using the ZORBAX column, with approximate elution times of 1.7 min for M4 and 2.1 min for HTL and ^2^H_4_-HTL. In higher concentration samples, a small interfering peak was detected on the HTL extracted ion channel at a retention time of 1.7 min; this was calculated to contribute ≤0.34% of the response in the HTL peak and was deemed insignificant. Chromatographic resolution enabled selective integration of the correct HTL peak at 2.1 min. In-source collision-induced dissociation may have caused partial deglucuronidation of the M4 metabolite, resulting in this interfering peak at 1.7 min.

**Figure 3.**
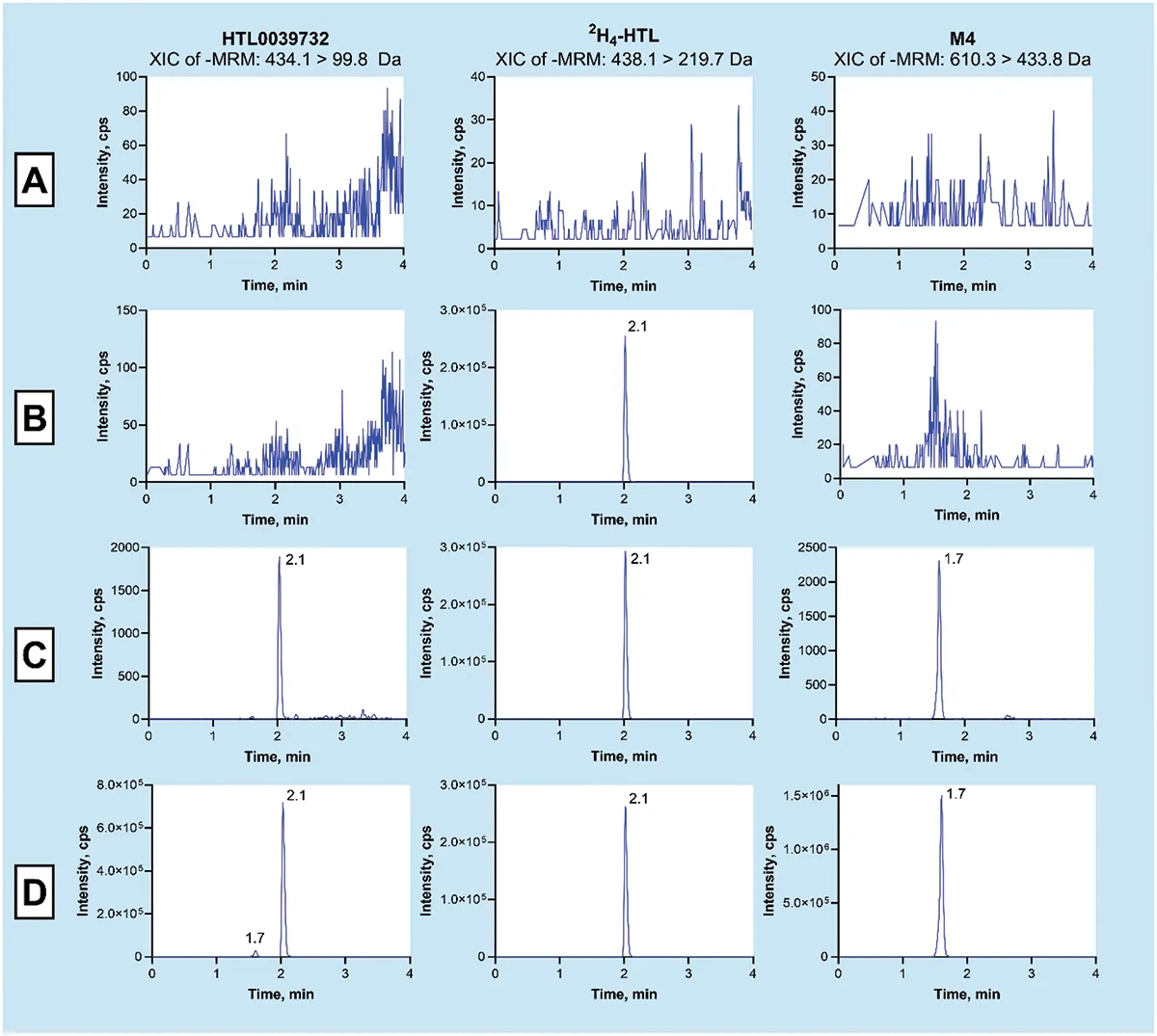
Representative chromatograms from a 10 µL on-column injection of (A) blank plasma, (B) blank plasma with internal standard, (C) a lower limit of quantification standard, and (D) a patient plasma sample taken 4 h after a 320 mg oral dose of HTL0039732.

Abbreviations: Cps: counts per second; Da: Daltons; Min: minutes; MRM: negative‑mode multiple reaction monitoring scan; XIC: extracted ion current.

Typical chromatograms are shown in Figure 3, demonstrating peak shape in A) blank plasma, B) blank plasma extracted with IS, C) an LLOQ standard, and D) a plasma sample taken from a clinical trial patient.

### 3.2. Method validation

#### 3.2.1. Limits of detection and quantification

The concentrations that gave S/N ratios of 3:1, to determine the LOD, were 2 ng/mL for HTL and 1 ng/mL for M4. LOQs with S/N ratios of 10:1 were achieved at 10 ng/mL and 5 ng/mL for HTL and M4, respectively (Supplementary Figure S2).

#### 3.2.2. Selectivity

The method was selective for both analytes and the IS, with mean responses in six sources of blank plasma as a percentage of the mean responses in the LLOQ of 3.05%, 1.07%, and 0.0370% for HTL, M4, and ^2^H_4_-HTL, respectively.

#### 3.2.3. Matrix effect

No matrix effects were observed in any of the six batches of blank plasma for either HTL or M4. The precision of IS-normalized matrix factor ranged from 1.29–13.0% and 6.99–11.4% for HTL and M4, respectively. The mean accuracy for the low, medium, and high QCs prepared across six batches of plasma ranged from 104–113% for HTL, with precision≤ 5.04%, and from 91.5–101% for M4, with precision ≤ 6.78%.

#### 3.2.4. Recovery

The mean recovery values for HTL were 48.1%, 49.7%, and 47.8% at concentrations of 30, 800, and 1600 ng/mL, respectively, with precision ≤ 10.8%. The method exhibited higher recovery for M4, with mean values of 85.5%, 81.5%, and 78.6% at concentrations of 15, 400, and 800 ng/mL, respectively, with precision ≤ 4.97%. The mean recovery of ^2^H_4_-HTL was 50.9% with precision of 8.67%. Although the recoveries between HTL and M4 were different, they both exhibited low precision, and thus were acceptable.

#### 3.2.5. Linearity and range

**Figure 4.**
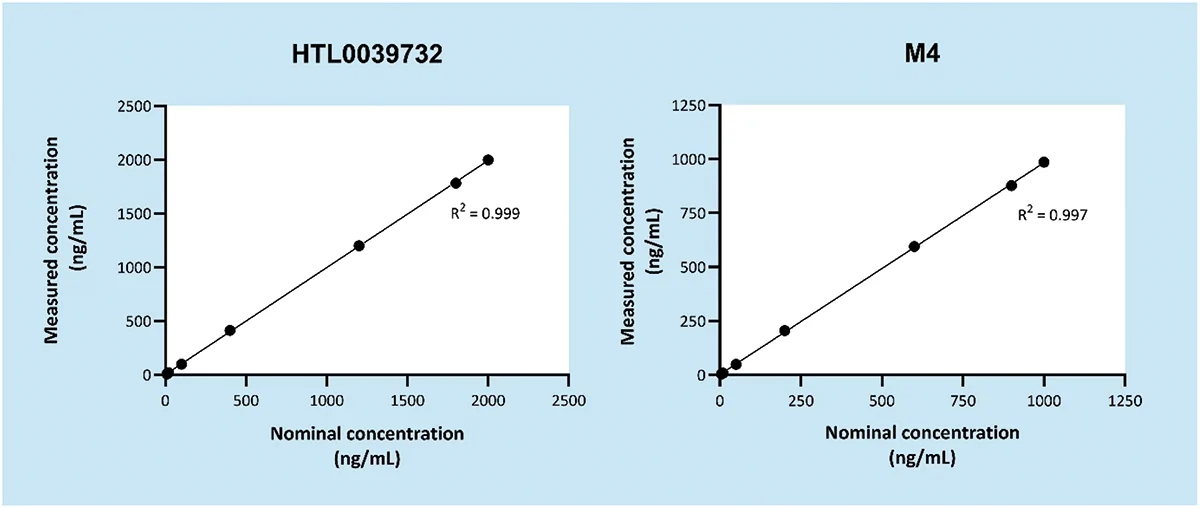
Mean calibration points over seven concentrations of HTL0039732 (10–2000 ng/mL) and M4 (5–1000 ng/mL), from five separate experiments, using a linear regression weighting of 1/x^2^. R^2^: coefficient of determination.

Reproducible linearity was observed between five independent analytical runs by analyzing standard curves across the range of 10–2000 ng/mL for HTL and 5–1000 ng/mL for M4. A linear regression weighting of 1/x^2^ gave the highest mean r^2^ values across the five runs: 0.999 for HTL and 0.997 for M4. Representative standard curves are displayed in Figure 4 and inter-day standard curve precision data are shown in Table 1 and Supplementary Table S1.

**Table 1.** Calibration curve statistics for HTL0039732 across five independent analytical runs, showing the mean calculated concentrations of seven calibration points between 10 and 2000 ng/mL, analyzed in duplicate per concentration.

Day	Nominal concentration (ng/mL)							Linear equation parameters		
	10.0	20.0	100	400	1200	1800	2000	m	c	R^2^
1	10.4	18.6	97.3	401	1241	1799	2048	0.00196	0.00127	0.999
2	10.2	19.1	100	413	1188	1761	2040	0.00200	0.00420	0.999
3	10.0	21.7	99.0	421	1191	1784	1950	0.00196	0.00848	0.999
4	10.1	19.6	101	409	1199	1787	1966	0.00210	0.00983	1.00
5	10.0	20.1	97.8	419	1183	1780	1995	0.00206	0.00854	0.998
Mean	10.1	19.8	99.1	413	1200	1782	2000	0.00202	0.00646	1.00
SD	0.156	1.19	1.68	7.95	23.4	13.8	43.3			
Accuracy (%)	101	99.1	99.1	103	100	99.0	100			
Precision (%)	1.54	6.02	1.70	1.93	1.95	0.776	2.17			

c: y-intercept; m: slope; R^2^: coefficient of determination; SD: standard deviation.

#### 3.2.6. Intra- and inter-day precision

Intra-day precision was assessed within one analytical run containing five QC levels (*n* = 8 per concentration). The precision for HTL was ≤11.8% and ≤8.62% for M4. The accuracy ranged from 81.1–100% for HTL and 90.6–102% for M4. Inter-day precision was assessed across five independent analytical runs, each containing four QC levels (*n* = 5 per concentration). The precision was ≤11.9% for HTL (Table 2) and ≤7.45% for M4 (Supplementary Table S2). The accuracy ranged from 86.0–101% for HTL and 88.6–103% for M4.

**Table 2.** Inter-day precision and accuracy of the analytical method for quantifying HTL0039732 in human plasma. Quality controls (QCs) were assessed across four levels: lower limit of quantification (LLOQ), low-quality control (LQC), mid-quality control (MQC), and high-quality control (HQC).

Sample	Day	HTL0039732 concentration (ng/mL)		Accuracy (%)	Precision (%)	HTL0039732 concentration (ng/mL)		Accuracy (%)	Precision (%)	RE (%)
		Mean	SD			Mean	SD			
LLOQ (10 ng/mL)	1	8.11	0.955	81.1	11.8					
	2	8.07	0.980	80.7	12.1					
	3	8.41	1.32	84.1	15.7					
	4	8.99	0.934	89.9	10.4					
	5	9.42	0.904	94.2	9.59					
	1–5					8.60	1.02	86.0	11.9	−14.0
LQC (30 ng/mL)	1	29.9	1.82	100	6.09					
	2	27.8	1.45	92.7	5.20					
	3	28.8	1.73	96.1	5.99					
	4	30.0	1.17	100	3.89					
	5	30.5	1.25	102	4.10					
	1–5					29.4	1.48	98.0	5.05	−2.00
MQC (800 ng/mL)	1	799	18.7	100	2.34					
	2	803	22.9	100	2.85					
	3	802	16.7	100	2.08					
	4	817	15.5	102	1.90					
	5	828	16.1	104	1.94					
	1–5					810	18.0	101	2.22	1.25
HQC (1600 ng/mL)	1	1587	38.7	99.2	2.44					
	2	1557	43.2	97.3	2.77					
	3	1568	37.7	98.0	2.40					
	4	1603	28.5	100	1.78					
	5	1601	50.3	100	3.14					
	1–5					1583	39.6	98.9	2.51	−1.06

Abbreviations: HQC: High-quality control; LLOQ: Lower limit of quantification; LQC: Low-quality control; MQC: Mid-quality control; RE: Relative error.

#### 3.2.7. Anticoagulant effect

There were no observable differences in the quantification of HTL or M4 in QCs prepared from plasma containing Na-citrate, Li-heparin, or K_2_-EDTA, suggesting that the use of any of these anticoagulants in a clinical setting would be appropriate. The accuracy ranged from 97.5–107% for HTL and 91.2–102% for M4. The precision was ≤5.09% for HTL and ≤5.11% for M4 (Table 3 and Supplementary Table S3).

**Table 3.** Short- and long-term stability and anticoagulant effect data for HTL0039732 (HTL).

Condition	Matrix	Time	LQC (30 ng/mL)						HQC (1600 ng/mL)					
			Mean	SD	Accuracy (%)	Precision (%)	T0 mean (ng/mL)	Difference from T0 (%)	Mean	SD	Accuracy (%)	Precision (%)	T0 mean (ng/mL)	Difference from T0 (%)
RT	Plasma	7 h	30.9	1.57	103	5.08	30.5	1.35	1670	44.1	104	2.64	1601	4.32
-20°C	Plasma	12 months	33.3	0.59	111	1.77	29.9	11.3	1763	23.1	110	1.31	1587	11.1
4°C	Extract	24 h	30.0	1.01	99.8	3.36	30.5	−1.80	1599	56.0	99.9	3.50	1601	−0.19
Anticoagulant	Na Citrate Plasma		32.2	1.64	107	5.09			1606	66.2	100	4.12		
	Li Heparin Plasma		29.5	1.36	98.2	4.60			1559	23.7	97.5	1.52		
	K_2_ EDTA Plasma		29.3	1.25	97.6	4.27			1578	40.8	98.6	2.58		

Abbreviations: CV: Coefficient of variation; EDTA: Ethylenediaminetetraacetic acid; HQC: High quality control; LQC: Low quality control; RT: Room temperature.

Data show the mean, accuracy, and precision of low and high-quality controls (QCs) under room‑temperature, frozen, and refrigerated storage conditions and the percentage change in mean concentration from the initial analysis immediately after preparation. Anticoagulant data show the accuracy and precision of the QCs prepared in plasma containing Na-citrate, Li-heparin, and K2-EDTA anticoagulants.

#### 3.2.8. Dilution integrity

All dilution methods were deemed to be acceptable for diluting patient samples with high concentrations of analyte into the analytical range. Across all dilution factors, accuracy ranged from 91.8–98.5% and 94.3–101%, and precision was ≤9.50% and ≤11.7%, for HTL and M4, respectively.

#### 3.2.9. Stability

The stability of HTL and M4 was established across several conditions by preparing low and high QCs, analyzing immediately, and repeating analysis following relevant storage conditions to establish a percentage difference. HTL and M4 were stable in plasma for 7 h at room temperature and 12 months at −20°C and in extracted supernatant for 24 h at 4°C. The difference from the T0 test was ≤11.3% for HTL and ≤7.67% for M4 across all test conditions (Table 3 and Supplementary Table S3).

Preclinical method validation proved the stability of HTL and M4 in human plasma over a minimum of five freeze–thaw cycles. These results show that the anticipated storage conditions of patient samples during clinical evaluation will not affect the quantitative analysis.

#### 3.2.10. Carryover of the analyte and IS

The method displayed acceptable levels of carryover for both analytes and ^2^H_4_-HTL. The mean responses in the blank plasma, as a percentage of the mean responses in the LLOQ, were 12.2%, 5.45% and 0.0703% for HTL, M4, and ^2^H_4_-HTL, respectively.

#### 3.2.11. Patient sample analysis

**Figure 5.**
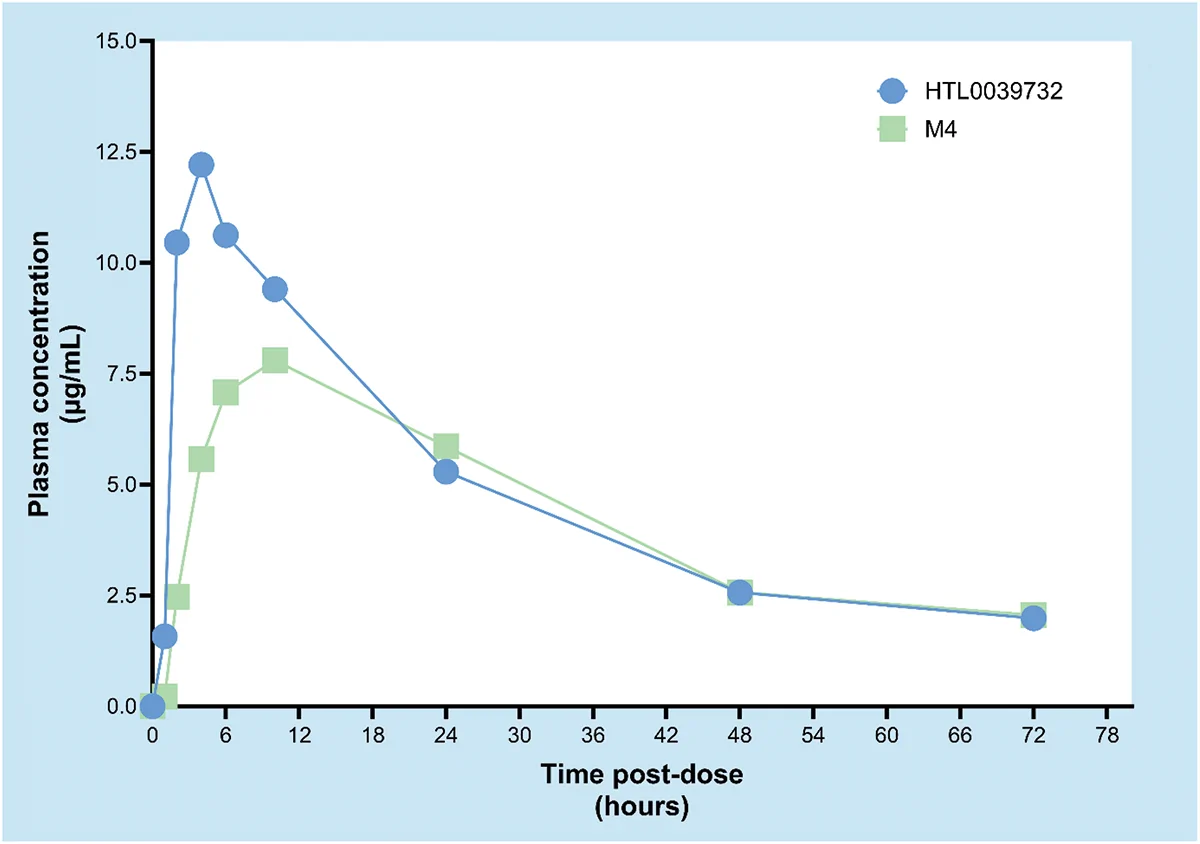
Representative plasma concentration–time profile for HTL0039732 and M4 in a patient following a single 320 mg oral dose of HTL0039732. Pharmacokinetic sampling took place pre-dose and 1, 2, 4, 6, 10, 24, 48, and 72 h post-dose.

The method has successfully been applied to the analysis of plasma samples taken from clinical trial patients following oral doses between 80 mg and 640 mg of HTL per day. Representative plasma concentration–time data from a patient who received an initial dose of 320 mg are shown in Figure 5. In this patient, plasma concentrations of HTL and M4 were measured over a 72-h period following the dose, with C_max_ values of 12.2 µg/mL and 7.80 µg/mL, respectively. Specific chromatograms for each analyte were obtained following extraction of the patient plasma samples (Figure 3(D)).

## 4. Discussion

Quantitative assessment of novel drugs represents a key element of first-in-human clinical trials. Such assessments require analytical methods that can stand up to repeated use across the course of a trial. Here, we have described the development and validation of a robust, sensitive LC-MS/MS method for the quantification of the novel anticancer therapeutic, HTL. This paper describes the first validated method to measure concentrations of HTL and its metabolite, M4, in human plasma.

Accurate results were consistently reproducible across the analytical range between analyses on multiple days. The method exhibited consistent recovery, good selectivity, and was not impacted by matrix effects, anticoagulant effects, or carryover. In addition, the stability of HTL and M4 was proven across a set of relevant conditions and durations, including room temperature, refrigerated, and frozen storage. Investigations into potential interference of co-administered drugs were not carried out as part of the assay method validation as it was felt unlikely that co-administered drugs with contrasting chemical structures would interfere with the assay. Also, such an approach would be challenging due to the large number of potentially concomitant medications being prescribed to late-stage cancer patients participating in an early‑phase clinical trial.

The validation of this method enabled confident application to patient sample analysis during a Phase I/IIa clinical trial investigating the safety, tolerability, and pharmacokinetics of HTL in patients with advanced solid tumors. The trial recruited 35 patients to Phase I, where data generated by application of this method were used to inform dosing decisions and have been used to determine a RP2D of 160 mg/day.

Treatment-related adverse events (TRAEs) were reported in 77% of trial patients during Phase I; however, only 14% experienced a grade 3+ TRAE, and there were no reported grade 4 or 5 TRAEs [11]. Partial responses were observed in two patients treated at a HTL dose of 160 mg in combination with atezolizumab, and stable disease was reported in 77% of patients. These preliminary results appear tentatively favorable relative to other EP4 antagonists currently undergoing early‑phase trials. Phase I trials of vorbipiprant and ONO-4578 have reported progressive disease in 46% and 77%, respectively, of patients treated with the investigative medicinal product (IMP) combined with anti-PD-1 therapy [12,13]. However, in the context of efficacy data generated during Phase I cancer trials, which recruit approximately 30 refractory, heavily pre-treated patients with advanced metastatic disease, efficacy data must be solidified by Phase II and Phase III studies with larger cohorts of clinically relevant patients.

The RP2D of HTL was 160 mg, once daily, which can be compared to other IMPs in the EP4 antagonist class; DT-9081, AN0025, and E7046 have RP2Ds of 500–600 mg per day [14–16], suggesting a lower dose of HTL may be sufficient to achieve systemic concentrations yielding therapeutically relevant receptor occupancy. In the current HTL early‑phase clinical trial, doses ≥160 mg were shown to maintain >90% EP4 receptor inhibition over the dosing interval for 95% of patients.

Pharmacokinetics form the bedrock of early‑phase clinical trials, allowing the determination of optimal dosing strategies, safety, tolerability, and efficacy in the context of the drug’s concentration within the body. Analytical methods to determine drug concentration are, therefore, imperative to the success of each trial. The method described in this paper will continue to be employed as HTL advances through the drug development process.

## 5. Conclusion

Here, we have described the development and validation of a robust and sensitive LC-MS/MS method for the quantification of HTL and M4 in human plasma using ^2^H_4_-HTL as an internal standard. The method has been successfully applied to the analysis of patient PK samples in a clinical setting and will continue to support the first-in-human trial of HTL for the treatment of advanced solid tumors. The resulting PK data were used to inform the RP2D. The inclusion of M4 in the PK measurements has enabled investigations into whether this acyl glucuronide metabolite may be reverted to HTL via de-glucuronidation and subsequently reabsorbed and recirculated.

## Supplementary Material

Heptares Supplementary Material Bioanalysis Submission.docx

## Data Availability

The data that support the findings of this study are available from the corresponding author (GV) upon reasonable request.

## Article highlights

**Background:**

- A LC-MS/MS method was developed and validated to quantify HTL0039732, a novel EP4 antagonist, and its acyl glucuronide metabolite HTL0065658 (M4).

**Experimental:**

- Acetonitrile, spiked with deuterium-labeled internal standard, was used as a protein precipitation solvent to extract analytes from plasma.

**Results and discussion:**

- A novel LC-MS/MS method to quantify HTL0039732 and M4 in human plasma was developed and validated according to EMA and FDA bioanalytical method validation guidelines.
- Linear ranges of 10–2,000 ng/mL of HTL0039732 and 5–1,000 ng/mL of M4 were achieved.
- The method described is being utilized for the analysis of patient samples in an ongoing Phase I/IIa clinical trial investigating HTL0039732.
