# Supplementary material for: Development and validation of a LC-MS/MS method for the quantification of the E-type prostanoid receptor 4 antagonist HTL0039732 in human plasma, for a Phase I/IIa clinical trial
Source: Bioanalysis. 2026 Aug 6;18(11):981–90. doi: 10.1080/17576180.2026.2712785 (PMC13463273; doi:10.1080/17576180.2026.2712785)
Supplement: Supplementary file 1 [file IBIO_A_2712785_SM7282.docx]

**Figure S1**

**Figure S2**

| **Day** | **Nominal Concentration (ng/mL)** | | | | | | | **Linear Equation Parameters** | | |
| --- | --- | --- | --- | --- | --- | --- | --- | --- | --- | --- |
|  | 5.00 | 10.0 | 50.0 | 200 | 600 | 900 | 1000 | m | c | R² |
| **1** | 5.15 | 8.424 | 45.1 | 188 | 605 | 860 | 971 | 0.00674 | 0.00292 | 0.992 |
| **2** | 5.14 | 8.394 | 51.9 | 210 | 560 | 908 | 1004 | 0.00583 | 0.0103 | 0.998 |
| **3** | 5.01 | 10.0 | 48.9 | 208 | 609 | 890 | 979 | 0.0102 | 0.00995 | 0.999 |
| **4** | 4.95 | 10.2 | 49.9 | 208 | 600 | 861 | 996 | 0.0108 | 0.0110 | 0.999 |
| **5** | 5.08 | 9.62 | 50.7 | 212 | 601 | 870 | 981 | 0.0104 | 0.0147 | 0.999 |
| **Mean** | 5.07 | 9.32 | 49.3 | 205 | 595 | 878 | 986 | 0.00879 | 0.00977 | 0.997 |
| **SD** | 0.0841 | 0.853 | 2.60 | 9.67 | 19.9 | 20.8 | 13.5 |  |  |  |
| **Accuracy (%)** | 101 | 93.2 | 98.6 | 103 | 99.2 | 97.5 | 98.6 |  |  |  |
| **Precision (%)** | 1.66 | 9.16 | 5.27 | 4.71 | 3.35 | 2.37 | 1.37 |  |  |  |

**Table S1**

**Table S2**

| **Sample** | **Day** | **HTL-M4 concentration (ng/mL)** | | **Accuracy (%)** | **Precision (%)** | **HTL-M4 concentration (ng/mL)** | | **Accuracy (%)** | **Precision (%)** | **RE (%)** |
| --- | --- | --- | --- | --- | --- | --- | --- | --- | --- | --- |
|  |  | **Mean** | **SD** |  |  | **Mean** | **SD** |  |  |  |
| **LLOQ (5 ng/mL)** | 1 | 4.53 | 0.930 | 90.6 | 8.62 |  |  |  |  |  |
|  | 2 | 4.23 | 0.200 | 84.5 | 4.74 |  |  |  |  |  |
|  | 3 | 4.11 | 0.260 | 82.3 | 6.31 |  |  |  |  |  |
|  | 4 | 4.79 | 0.336 | 95.7 | 7.03 |  |  |  |  |  |
|  | 5 | 4.48 | 0.475 | 89.7 | 10.6 |  |  |  |  |  |
|  | 1-5 |  |  |  |  | 4.43 | 0.332 | 88.6 | 7.45 | -11.4 |
| **LQC (15 ng/mL)** | 1 | 14.7 | 0.598 | 98.2 | 4.06 |  |  |  |  |  |
|  | 2 | 13.9 | 0.589 | 92.6 | 4.24 |  |  |  |  |  |
|  | 3 | 13.9 | 0.477 | 92.8 | 3.43 |  |  |  |  |  |
|  | 4 | 16.5 | 0.708 | 110 | 4.28 |  |  |  |  |  |
|  | 5 | 15.2 | 1.09 | 102 | 7.19 |  |  |  |  |  |
|  | 1-5 |  |  |  |  | 14.8 | 0.693 | 98.7 | 4.64 | -1.33 |
| **MQC (400 ng/mL)** | 1 | 407 | 10.5 | 102 | 2.59 |  |  |  |  |  |
|  | 2 | 396 | 11.0 | 99.0 | 2.78 |  |  |  |  |  |
|  | 3 | 397 | 7.57 | 99.2 | 1.91 |  |  |  |  |  |
|  | 4 | 445 | 11.6 | 111 | 2.61 |  |  |  |  |  |
|  | 5 | 407 | 13.0 | 102 | 3.19 |  |  |  |  |  |
|  | 1-5 |  |  |  |  | 410 | 10.7 | 103 | 2.61 | 2.50 |
| **QC (800 ng/mL)** | 1 | 792 | 33.1 | 99.0 | 4.17 |  |  |  |  |  |
|  | 2 | 774 | 17.0 | 96.7 | 2.20 |  |  |  |  |  |
|  | 3 | 783 | 18.7 | 97.8 | 2.39 |  |  |  |  |  |
|  | 4 | 869 | 28.1 | 109 | 3.23 |  |  |  |  |  |
|  | 5 | 791 | 51.7 | 98.9 | 6.53 |  |  |  |  |  |
|  | 1-5 |  |  |  |  | 802 | 29.7 | 100 | 3.70 | 0.250 |
| HQC: High quality control; LLOQ: Lower limit of quantification; LQC: Low quality control; MQC: Mid quality control; RE: Relative error | | | | | | | | | | |

| **Condition** | **Matrix** | **Time** | **LQC (15 ng/mL)** | | | | | |  | **HQC (800 ng/mL)** | | | | | |
| --- | --- | --- | --- | --- | --- | --- | --- | --- | --- | --- | --- | --- | --- | --- | --- |
|  |  |  | **Mean** | **SD** | **Accuracy  (%)** | **Precision  (%)** | **T0 mean  (ng/mL)** | **Difference from T0 (%)** |  | **Mean** | **SD** | **Accuracy  (%)** | **Precision  (%)** | **T0 mean  (ng/mL)** | **Difference from T0 (%)** |
| **RT** | Plasma | 7 hours | 14.0 | 0.324 | 93.6 | 2.31 | 15.2 | -7.67 |  | 760 | 19.7 | 95.0 | 2.31 | 791 | -3.99 |
| **-20°C** | Plasma | 12 months | 14.9 | 0.387 | 99.4 | 2.59 | 14.7 | 1.47 |  | 750 | 14.4 | 93.8 | 1.92 | 792 | -5.31 |
| **4°C** | Extract | 24 hours | 14.5 | 0.817 | 96.6 | 5.64 | 15.2 | -4.72 |  | 785 | 38.1 | 98.1 | 4.85 | 791 | -0.786 |
| **Anticoagulant** | Na Citrate Plasma |  | 15.0 | 0.768 | 100 | 5.11 |  |  |  | 762 | 29.9 | 95.2 | 3.93 |  |  |
|  | Li Heparin Plasma |  | 13.7 | 0.337 | 91.2 | 2.46 |  |  |  | 816 | 23.0 | 102 | 2.82 |  |  |
|  | K_2_ EDTA Plasma |  | 15.1 | 0.631 | 101 | 4.17 |  |  |  | 799 | 12.6 | 99.9 | 1.58 |  |  |
| CV: Coefficient of variation; EDTA: Ethylenediaminetetraacetic acid; HQC: High quality control; LQC: Low quality control; RT: Room temperature | | | | | | | | | | | | | | | |

**Table S3**
